# Effectiveness of Percutaneous Balloon Mitral Valvuloplasty for Rheumatic Mitral Stenosis with Mild to Severe Mitral Regurgitation

**DOI:** 10.1155/2016/3298343

**Published:** 2016-02-29

**Authors:** LinXiang Lu, Lang Hong, Jun Fang, LiangLong Chen

**Affiliations:** ^1^Department of Cardiology, Union Hospital, Fujian Medical University, No. 29, Xinquan Road, Fuzhou, Fujian 350001, China; ^2^Department of Cardiology, The People's Hospital of Jiangxi Province, No. 92, Aiguo Road, Nanchang, Jiangxi 330006, China

## Abstract

This study is designed to test whether percutaneous balloon mitral valvuloplasty (PBMV) is effective for rheumatic mitral stenosis in Chinese patients with moderate to severe mitral regurgitation. Fifty-six patients with rheumatic mitral valve stenosis were divided into the mild, moderate, and severe regurgitation groups. Cardiac ultrasonography was measured before and 1 to 2 days after PBMV. Following PBMV, the mitral orifice was enlarged, and the left atrial diameter was reduced in the 3 patient groups. The enlargement of the mitral orifice in the mild regurgitation group was greater than that observed in the moderate and severe regurgitation groups. The size of the regurgitation area increased in the mild regurgitation group and decreased in the moderate and severe regurgitation groups, with the decrease in the severe regurgitation group being greater than that in the moderate regurgitation group. Therefore, PBMV is effective for treating rheumatic mitral stenosis in Chinese patients with mild to severe mitral regurgitation.

## 1. Introduction

Rheumatic heart disease (RHD) is a progressive and chronic condition caused by complement-mediated damage to the atrioventricular valves that occurs as a result of the inflammatory response in rheumatic fever [1, 2]. The worldwide prevalence and annual incidence of RHD have been estimated to be >15 million cases and >280 000 cases per year, respectively, resulting in an annual mortality of approximately 220 000 deaths per year [3]. Although the incidence of RHD has decreased in developed nations in recent decades [4], it remains a serious public health problem in developing regions throughout the world [1, 3, 5]. The prevalence of RHD in Chinese adults has been estimated to be approximately 2% [6].

The mitral valve is often the structure that is most highly affected by the verruciform fibrotic lesions that are characteristic of RHD pathology, leading to mitral valve stenosis (MS) and, in some cases, mitral valve regurgitation (MR) due to incomplete closure of the mitral valve during ventricular systole. Percutaneous balloon mitral valvuloplasty (PBMV) has been developed for treating rheumatic MS in patients with favorable valvular conditions as an alternative for surgical interventions [7, 8]. With a success rate of approximately 93% and lower rates of complications and postoperative infection, compared to closed mitral commissurotomy, PBMV has become the treatment of choice for rheumatic MS [9, 10].

Previous studies of PBMV for MS have often excluded patients with moderate to severe MR [11]. Therefore, the curative effects of PBMV in patients with rheumatic MS and moderate to severe MR remain largely unclear. In addition, studies of PBMV in Chinese patients with rheumatic MS are scant. The aim of our current study was to evaluate the effectiveness of PBMV for rheumatic MS in Chinese patients with mild to severe MR.

## 2. Patients and Methods

### 2.1. Patients

Fifty-six RHD patients who underwent PBMV for rheumatic MS at Union Hospital (Fuzhou, Fujian, China) or the People's Hospital of Jiangxi Province (Nanchang, Jiangxi, China) between May 2008 and June 2012 were included in our study. Informed consent was obtained from each patient before our study was conducted. Cardiac ultrasonography was performed, and the patients were divided into 3 groups based on the size of the area across which MR was detected (MR area), with a larger MR area representing more severe MR. The mild MR group consisted of patients with an MR area <4 cm^2^ (*n* = 30). Patients with an MR area of 4 to 8 cm^2^ were assigned to the moderate MR group (*n* = 10), and those with an MR area >8 cm^2^ were assigned to the severe MR group (*n* = 16). Our study was approved by the Biomedical Ethics Committee of Fujian Medical University.

### 2.2. Procedure

All of the patients underwent PBMV using an Inoue balloon catheter (Jiangxi Yikang Medical Instrument Group Co., Ltd., Nanchang, China ). The waist diameter of the balloon and the pressure in the left atrium were monitored throughout the procedure. The balloon was placed in the mitral valve orifice (MVO), and 19 mL of 76% meglumine (Shanghai Xudong Haipu Pharmaceutical Co., Ltd., China) was used to inflate the balloon, after which meglumine was injected in 0.5 to 1.0 mL increments to gradually increase the balloon diameter to a maximum of 23 to 24 mm. The balloon diameter was initially calculated as height (cm) + 100/10, but for the moderate and severe regurgitation groups, the balloon was inflated until the left atrial pressure had dropped by >50%, the average pressure in the left atrium was <15 mm Hg (1.995 kPa), or the crescendo diastolic murmur of the mitral valve had weakened substantially or had become undetectable. All patients were treated with multiple balloon inflations. Cardiac ultrasonography was used to measure the cross-sectional area of the MVO (MVO area), the MR area, and the diameter of the left atrium of each patient at 1 to 2 days following PBMV.

### 2.3. Statistical Analysis

All of the statistical analyses were performed using the SPSS, version 17.0, software (IBM, Armonk, NY, USA). Continuous variables are expressed as the mean ± standard deviation. For continuous variables, intergroup differences between 2 groups were evaluated using *t*-tests, and those between 3 groups were evaluated using a one-way analysis of variance. For categorical variables, intergroup differences were evaluated using the Chi-squared test. All of the tests were two-tailed, and the level of statistical significance was set at *P* < 0.05.

## 3. Results

### 3.1. Patient Characteristics

Our rheumatic MS cohort consisted of 15 men and 41 women (Table 1). The patients had a mean age of 47.88 ± 11.94 years (Table 1). No significant difference in sex or age was observed between the various MR groups (*P* > 0.05 for both).

### 3.2. Curative Effects of PBMV

Following PBMV, the size of the MVO area increased in all of the MR groups (*P* < 0.001, Table 2). The increase in the size of the MVO area in the mild MR group (0.84 ± 0.35 cm^2^) was significantly greater than that observed in the moderate and severe MR groups (0.62 ± 0.29 and 0.63 ± 0.22 cm^2^; *P* < 0.05 for both, Table 2). The increase in the size of the MVO area in the severe MR group was not significantly different from that in the moderate MR group (*P* > 0.05, Table 2). The diameter of the left atrium decreased significantly in the mild, moderate, and severe MR groups following PBMV (*P* = 0.002, *P* = 0.009, and *P* = 0.025, resp., Table 3). Although the preoperative diameter of the left atrium in the severe MR group was significantly greater than that in the mild group (*P* < 0.05), no significant difference in left atrial diameter was observed among the 3 MR groups after PBMV (*P* > 0.05), and the decreases in atrial diameter were statistically similar among the various MR groups (*P* > 0.05).

### 3.3. Effect of PBMV on MR

The size of the MR area in the mild MR group increased significantly following PBMV (−2.01 ± 3.64 cm^2^, *P* = 0.001, Table 4). By contrast, PBMV significantly reduced the size of the MR area in the moderate and severe MR groups (*P* = 0.010 and *P* = 0.008, resp.), and the decrease in the severe MR group (2.14 ± 2.91 cm^2^) was significantly greater than that in the moderate MR group (1.82 ± 1.88 cm^2^; *P* < 0.05, Table 4).

## 4. Discussion

Occurring as a late sequela in rheumatic fever, rheumatic MS is caused by fibrotic lesions resulting from the deposition of immune complexes and complement on the surface of the mitral valve [2, 3]. These structural abnormalities impede diastolic filling of the left ventricle, which reduces preload and increases the systolic pressure and the diastolic volume of the left atrium [2, 3]. Patients with rheumatic MS present with progressive dyspnea because reduced cardiac output results in pulmonary arterial hypertension, the effects of which are manifested as congestive heart failure in severe cases [12, 13]. Rheumatic valvular disease can also cause MR due to incomplete closure of the mitral valve during ventricular systole, which reduces cardiac output further [2, 8].

In China and many developing countries around the world, RHD remains a significant public health problem, with high morbidity and mortality [1, 4–6]. The use of PBMV for the treatment of rheumatic MS has become common practice worldwide [7, 8, 11], and in our current study in China, we found that PBMV was both a practical and effective treatment for such patients.

Though in previous studies open mitral valvuloplasty or mitral valve replacement was recommended [3, 5, 14, 15], our results showed that surgery should not be considered as a replacement for PBMV in patients with MS and severe mitral regurgitation.

In our Chinese cohort of rheumatic MS patients, PBMV effectively reduced the mean diameter of the left atrium and increased the mean cross-sectional MVO area in all of the rheumatic MS patient groups. The increase in MVO area was greater in patients with mild MR compared to those with moderate to severe MR, because the mild MR group can tolerate eventually larger balloon diameter.

Our results showed that PBMV reduced the severity of mitral regurgitation in Chinese patients, which is in accordance with previous reports of PBMV in patients with RHD [14, 16, 17]. However, the extent of MR actually decreased in those with moderate to severe MR as compared to those with mild MR. The explanation for this phenomenon appears to be multifactorial. First, since LAD (left atrial diameter) in the severe MR group was greater than in the other groups before PBMV, the relative narrowness of the mitral valve annulus may be at a greater extent due to more reduction of LAP (left atrial pressure) after PBMV. Another possible reason for a decrease in the severity of MR after PBMV in the moderate and severe MR groups may be related to commissural splitting. Before PBMV, the papillary muscles and tendons are shortened and adherent to the valve. Thus, the valve leaflets cannot open adequately in diastole or rise properly to close in systole. After successful PBMV, fused commissures are separated, and the mitral valve becomes relatively more mobile, permitting better closure during systole. In contrast, an increase in MR after PBMV appears to be related to rupture of the mitral valve leaflets rather than commissural splitting [18]. Moreover, the use of our rather cautionary balloon-sizing when performing PBMV (a stepwise dilatation technique) in the presence of more than mild MR avoids balloon oversizing and may have contributed to the low risk of severe mitral regurgitation in this study [19].

Despite avoiding surgical trauma to the chordae tendineae and papillary musculature, previous studies of the long term effects of PBMV for rheumatic MS have shown that the postoperative severity of MR may increase in some patients [17, 20]. We did not evaluate the long term effects of PBMV in our Chinese cohort. However, in our current study, none of the patients with mild or moderate MR developed severe MR immediately after the PBMV procedure. A limitation of our study was the limited number of mild moderate and severe MR patients and future studies with a larger sample size including long term effects of PBMV are warranted.

## Figures and Tables

**Table 1:** Comparison of sex and age in the 3 mitral regurgitation (MR) groups.

MR group	Sex (*n*)			Age (y)
	Men	Women	Total	
Mild	8	22	30	46.87 ± 13.39
Moderate	2	8	10	49.70 ± 8.47
Severe	5	11	16	49.16 ± 11.66
*P* value	0.8197			0.746

**Table 2:** Comparison of the cross-sectional area of the mitral valveorifice before and after percutaneous balloon mitral valvuloplasty in the 3 mitral regurgitation (MR) groups.

MR group	Preoperative area (cm^2^)	Postoperative area (cm^2^)	*P* value	Increase (cm^2^)
Mild	0.85 ± 0.22	1.69 ± 0.41	<0.0001	0.84 ± 0.35
Moderate	0.83 ± 0.18	1.45 ± 0.35	<0.0001	0.62 ± 0.29^#^
Severe	0.84 ± 0.21	1.45 ± 0.24^*∗*^	<0.0001	0.61 ± 0.26^*∗*^
*P* value	0.9771	0.0453		0.0254

^*∗*^ *P* < 0.05 for mild group versus severe group in the same column.

^#^ *P* < 0.05 for mild group versus moderate group in the same column.

**Table 3:** Comparison of the diameter of the left atrium before and after percutaneous balloon mitral valvuloplasty in the 3 mitral regurgitation (MR) groups.

MR group	Preoperative diameter (mm)	Postoperative diameter (mm)	*P* value	Reduction (mm)
Mild	47.93 ± 7.02	44.90 ± 7.23	0.002	3.03 ± 4.73
Moderate	49.70 ± 3.53	45.40 ± 4.77	0.009	4.30 ± 4.11
Severe	53.19 ± 8.91^*∗*^	48.94 ± 6.65	0.025	4.25 ± 6.78
*P* value	0.0463	0.1695		0.5529

^*∗*^ *P * < 0.05 for mild group versus severe group in the same column.

**Table 4:** Comparison of the mitral regurgitation area before and after percutaneous balloon mitral valvuloplasty in the 3 mitral regurgitation (MR) groups.

MR group	Preoperative area (cm^2^)	Postoperative area (cm^2^)	*P* value	Change (cm^2^)
Mild	1.22 ± 1.24	3.23 ± 3.92	0.001	−2.01 ± 3.64
Moderate	6.21 ± 0.87^#^	4.39 ± 2.25	0.010	1.82 ± 1.88
Severe	10.47 ± 1.73^*∗*c^	8.33 ± 3.62^*∗*a^	0.008	2.14 ± 2.91^*∗*b^
*P* value	<0.0001	0.0003		<0.0001

^*∗*^ *P* < 0.001 for mild group versus severe group in the same column.

^#^ *P* < 0.001 for mild group versus moderate group in the same column.

^a^ *P* < 0.05, ^b^ *P* < 0.01, and ^c^ *P* < 0.001 for moderate group versus severe group in the same column.
